# Trained immunity in recurrent *Staphylococcus aureus* infection promotes bacterial persistence

**DOI:** 10.1371/journal.ppat.1011918

**Published:** 2024-01-19

**Authors:** Xiao-Qi Lin, Zhen-Zhen Liu, Cheng-Kai Zhou, Liang Zhang, Yu Gao, Xue-Yue Luo, Jian-Gang Zhang, Wei Chen, Yong-Jun Yang

**Affiliations:** Department of Preventive Veterinary Medicine, College of Veterinary Medicine, Jilin University, Changchun, People’s Republic of China; University of Tubingen, GERMANY

## Abstract

Bacterial persister cells, a sub-population of dormant phenotypic variants highly tolerant to antibiotics, present a significant challenge for infection control. Investigating the mechanisms of antibiotic persistence is crucial for developing effective treatment strategies. Here, we found a significant association between tolerance frequency and previous infection history in bovine mastitis. Previous *S*. *aureus* infection led to *S*. *aureus* tolerance to killing by rifampicin in subsequent infection in vivo and in vitro. Actually, the activation of trained immunity contributed to rifampicin persistence of *S*. *aureus* in secondary infection, where it reduced the effectiveness of antibiotic treatment and increased disease severity. Mechanically, we found that *S*. *aureus* persistence was mediated by the accumulation of fumarate provoked by trained immunity. Combination therapy with metformin and rifampicin promoted eradication of persisters and improved the severity of recurrent *S*. *aureus* infection. These findings provide mechanistic insight into the relationship between trained immunity and *S*. *aureus* persistence, while providing proof of concept that trained immunity is a therapeutic target in recurrent bacterial infections involving persistent pathogens.

## Author summary

Skin and soft tissue infections (SSTIs), one of the most common chronic, relapsing, and refractory diseases for human and animals, are potentially involved in pathogen persistent infection. *Escherichia coli* tolerance frequency in bloodstream infection was associated with past infection, but little is known about how the previous episodes of infection affect bacterial persistency in subsequent challenges. In this study, we determined persister frequency was significantly associated with previous infection history in bovine mastitis. Using a trained immunity model, we uncovered trained immunity promoted *S*. *aureus* rifampicin persistency in secondary challenges, which might correlate with fumarate accumulation. We also demonstrated that combination therapy with metformin and rifampicin improved *S*. *aureus* clearance and disease severity. These data provide direct evidence that targeting trained immunity might be an effective strategy for recurrent diseases involving persistent pathogens.

## Introduction

*Staphylococcus aureus* (*S*. *aureus*) is the leading cause of skin and soft tissue infections (SSTIs), which are the primary portal of entry for life-threatening infections (e.g., bacteraemia and endocarditis) [1]. The recurrent *S*. *aureus* infections in children and adults occur in up to 50% within one year following SSTI [2,3]. Although frontline antibiotics are selected to treat *S*. *aureus* infection, clinical treatment failure and recurrent infection are still very frequent and poses a great threat to human public health and the ruminant breeding industry [4,5].

Antibiotic resistance is easily detected by the antibiotic susceptibility testing, but antibiotic persistence is still largely underestimated. Indeed, the development of antibiotic resistance has become a great global public health concern [6]. However, many patients with chronic bacterial infections often need long-lasting, cyclic administration of high doses of antibiotics treatment, even though the clinical isolates are susceptible [7]. These bacteria persist in biofilms, professional and nonprofessional cells or other niches to protect itself from being killed by active antibiotics [8].

Antibiotic persistence is defined as the ability of a small bacterial subpopulation to survive high bactericidal drug concentrations to which the bacteria are transient non-replicative and antibiotic-tolerant. Unlike antibiotic resistance, persistence is not genetically inherited. Persisters are usually characterized by biphasic kill curves and revert to a normal phenotype after antibiotic removal [9]. Recently, the Tolerance Disk (TD) test has offered an easy detection of bacterial tolerance or persistence in clinical isolates [10]. Several studies have shown that the high incidence of persisters in various bacterial infections, such as *Salmonella*, uropathogenic *Escherichia coli*, *Mycobacterium tuberculosis* as well as *S*. *aureus*, is a potential cause of chronic and recurrent infections, leading to eventual treatment failure [8,11–13]. This increases medical costs and potentially accelerates the emergence of drug-resistance [14]. Therefore, there is an urgent need to understand the mechanism of persistence of *S*. *aureus* more completely in order to develop new and effective treatment strategies.

A recent study reported that tolerant *E*. *coli* are frequently encountered among bloodstream isolates and are significantly associated with past infection [15]. High level of persisters was also found in clinical staphylococcal isolates [16]. Herein, we surveyed the prevalence of *S*. *aureus* persisters in mastitis cows and found the persister frequency is significantly associated with previous infection history (S1 **Table**). Prior *S*. *aureus* infection has been demonstrated to induce trained immunity to protect against subsequent infection, especially in recurrent *S*. *aureus* infection [17]. Trained immunity is the long-term functional reprogramming of innate immune cells after transient stimulation, resulting in an enhanced response to secondary challenge upon return to a non-activated state, which is a de facto innate immune memory [18].

In this study, we provide evidence for prior infection leads to the increased *S*. *aureus* persistence and enhanced innate immune response using a SSTIs model. We show that classical heat-killed *C*. *albicans* (HK-*C*. *a*)-induced trained immunity also facilitates *S*. *aureus* antibiotic persistence, which may be related to the accumulation of fumarate. As persister is a potential cause of treatment failure, we next treat recurrent *S*. *aureus* infection with metformin, a trained immunity inhibitor, in combination with antibiotics. We find metformin counteracts trained immunity-mediated persisters formation and ameliorates disease severity.

## Results

### Tolerance epidemiology of *S*. *aureus* in mastitis cows

**Fig 1:**
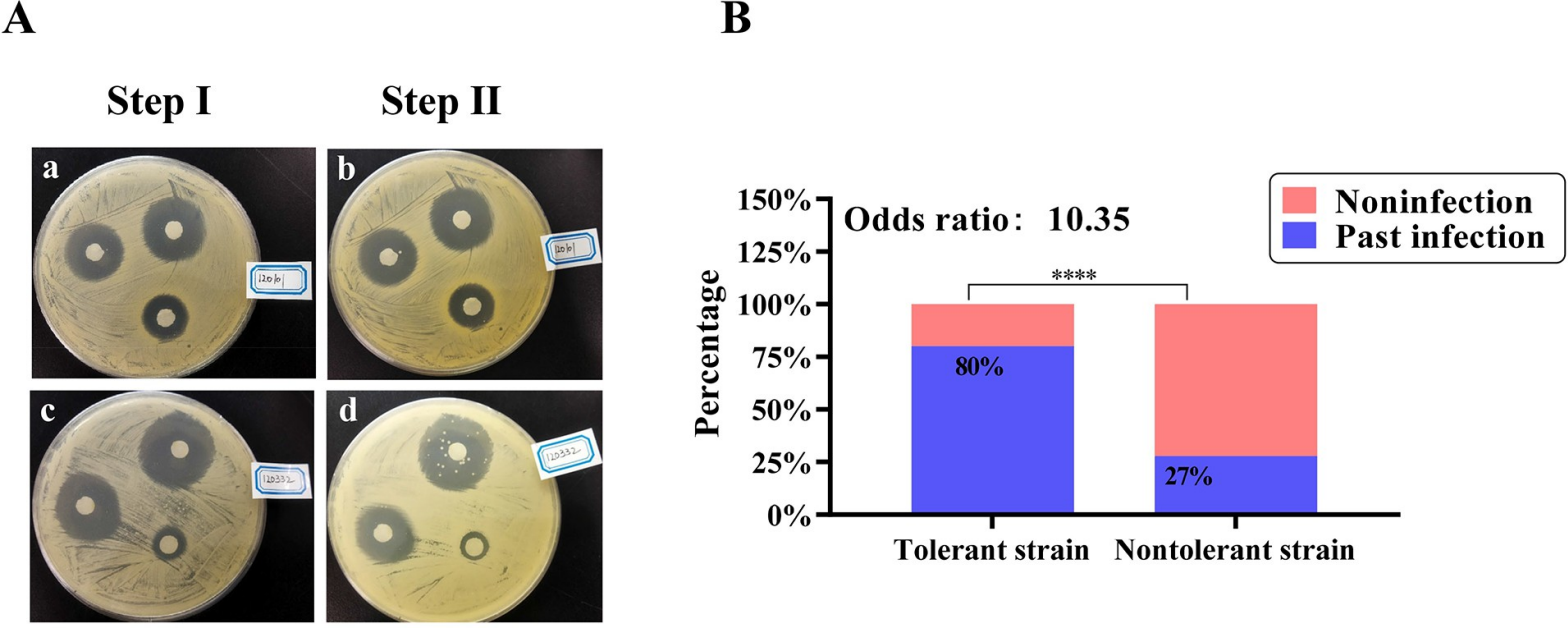
*S*. *aureus* tolerance in mastitis potentially associated with past infections. (A) Representative TDtest results. Two different strains from the retrospective cohort were plated on Mueller Hinton plates with 3 rifampicin discs (0.05, 0.5, and 5 μg) and incubated overnight. (a, c) 3 different inhibition zones appeared after the first incubation (small, medium, and large, respectively); (b, d) Addition of nutrients to the discs and second incubation reveal differences in tolerance level. (b), no late growth after second incubation. (d) Several colonies inside the inhibition zone indicated a high level of tolerance. TDtest, Tolerance Disk Test. (B) The correlation of past infection and bacterial tolerance. Odds ratio: 10.35, 95% Cl: 2.565 to 36.45. Data were analyzed by Fisher’s exact test.

Mastitis is one of the most serious SSTIs in lactating women and cows, ranging from acute clinical mastitis to chronic subclinical mastitis [19]. *S*. *aureus* is a major cause of acute mastitis and persists inside the udder in chronic persistent mastitis infection [8,20]. To investigate the correlation between *S*. *aureus* tolerance and past mastitis occurrence, we used a retrospective cohort of cows with mastitis to detect the epidemiology of tolerance in a clinical population. In total, we obtained 90 mastitis positive milk samples from the farm. Of the positive cultures, 76 (84%) grew *S*. *aureus*. The Tolerance Disk Test (TDtest) was performed as previously described [15]. Of milk isolates, 15 strains (15/76, 19.7%) were tolerant per the TDtest (**Fig 1A**), indicating a great tolerance prevalence. The baseline characteristics of cohort 1 mastitis cows were presented in **S1 Table**. Correlation analysis showed that cows with mastitis that had a higher tolerance index had a higher frequency of previous infection episodes **(Fig 1B)**.

### Antibiotic persistence is directly related to the innate immune protection of the host triggered by prior *S*. *aureus* infection

**Fig 2:**
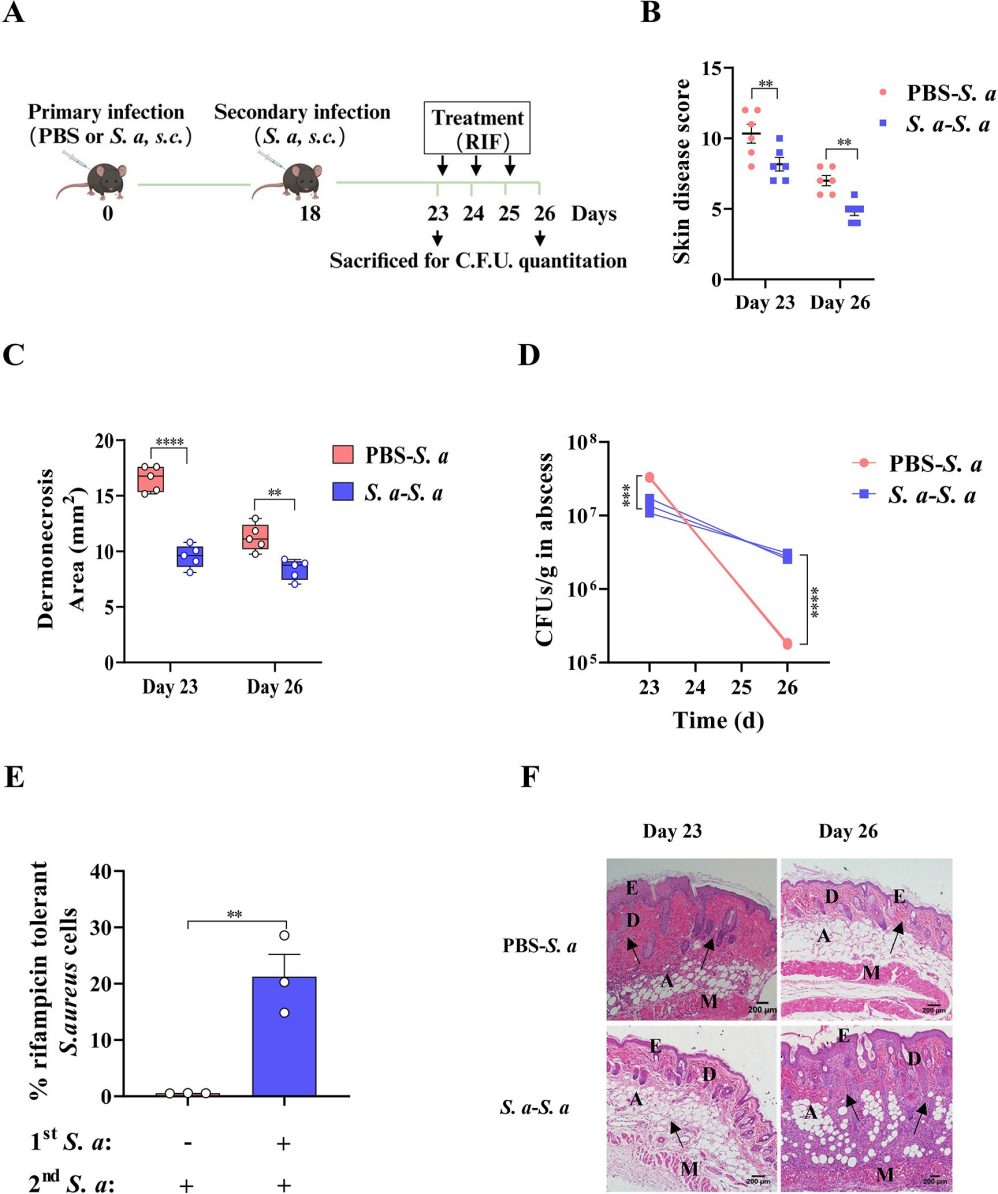
In vivo infection in mice demonstrates that antibiotic susceptibility of *S*. *aureus* is conversely correlated with prior infection. (A) Schematic representation. C57BL/6J mice were given subcutaneous (s.c.) primary infection of *S*. *aureus* or PBS 18 days prior to secondary *S*. *aureus* infection (s.c.). RIF therapy was initiated at 23 d and continued for 3 days. Created by Biorender. (B) Skin disease score of mice at 23 d and 26 d. (C) Skin lesion size of mice was measured at 23 d and 26 d. (D) Bacterial burden in skin abscesses from infected (*S*. *aureus*) or uninfected (PBS) mice was determined by CFU enumeration at 23 and 26 d. (E) % Survival of *S*. *aureus* cells in mice with different infection histories after rifampicin therapy compared with before rifampicin treatment (extrapolated from D). (F) Representative H&E-stained skin sections at 23 and 26 d (magnification of 100 ×). The black arrow indicates inflammatory cells infiltration. E: epidermis; D: dermis; A: adipose tissue; M: muscle fibres. *S*. *a*, *S*. *aureus*. Data pooled from 2 independent experiments with 5 to 6 mice per group. *p < 0.05, **p < 0.01, ***p < 0.001, *****p < 0.0001. Two-way ANOVA or student’s t test analysis of variance was performed and the data are presented as the means ± SEM.

To study the relationship between antibiotic tolerance and the host immune status in recurrent *S*. *aureus* infections, we compared the susceptibility of colonized *S*. *aureus* to rifampicin in mice with different infection histories **(Fig 2A)**. We found that prior *S*. *aureus* infected mice showed decreased skin lesions size and skin disease score in secondary skin infection regardless of rifampicin treatment **(Fig 2B and 2C)**. Bacteria within the abscess of mice with past *S*. *aureus* infection were more tolerant to rifampicin killing (about 39 folds) than the control mice **(Fig 2D and 2E)**. Histological analysis revealed epidermal hyperplasia, dermal thickening, disrupted dermal collagen fibres and severe inflammatory infiltration in the dermis and epidermis in the *S*. *aureus*-infected mice. Antibiotic treatment effectively alleviated the pathological damage and inflammatory cell infiltration in primary *S*. *aureus*-infected mice, but recurrent infection disturbed the rifampicin efficacy **(Fig 2F)**. These results indicated that previous *S*. *aureus* infection promotes bacterial tolerance to antibiotic killing during secondary *S*. *aureus* skin infection, which increases difficult to treat.

**Fig 3:**
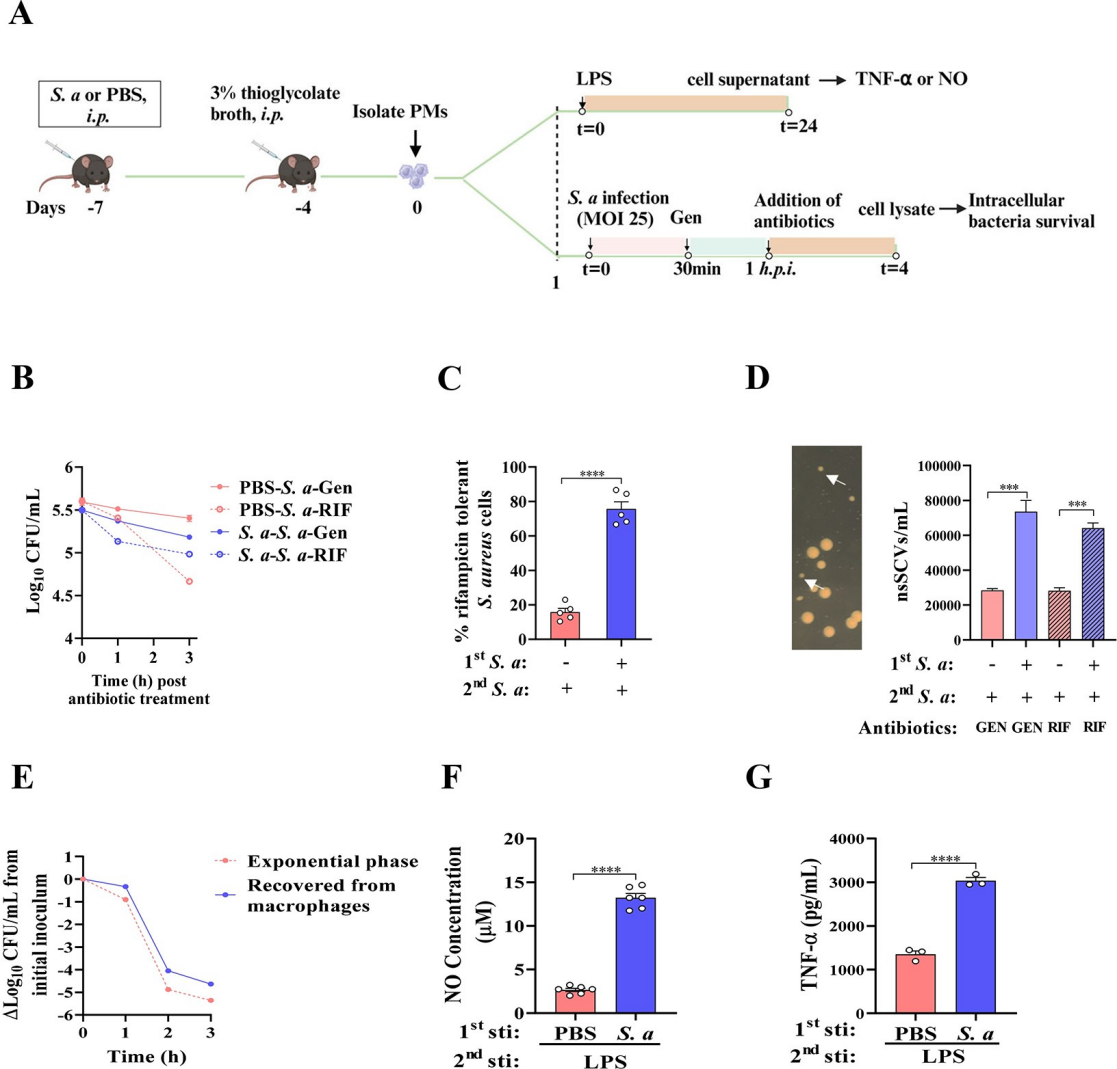
Previous *S*. *aureus* infection increases antibiotic persistence in host macrophages. (A) Schematic representation. The mice were infected intraperitoneally with *S*. *aureus* or PBS (i.p.) and 3 days later, 3% thioglycolate broth was injected. The peritoneal macrophages (PMs) were collected 4 d later and infected for 30 min with live *S*. *aureus* (MOI 25) next day. The plates were then washed and added gentamicin (100 μg/mL) for another 30 min to eliminate extracellular bacteria. At 1 h post infection, 8 μg/mL rifampicin (RIF) or 10 μg/mL gentamicin (control) was added and cell lysates were incubated on agar plates to quantify bacterial numbers (CFU). On the other hand, PMs were restimulated with LPS for 24 h and cell culture supernatant were obtained to detect NO and TNF-α. Created by Biorender. (B) Survival of *S*. *aureus* cells in PMs from uninfected (PBS) or infected (*S*. *aureus*) mice. (C) % Survival of *S*. *aureus* cells treated with rifampicin for 2 h compared to the gentamicin control (extrapolated from B). (D) The nonstable small colony variants (nsSCVs) morphology and abundance. The white arrow indicates nsSCVs, left panel. (E) Activity of RIF in broth against an exponential phase culture (red symbols) or bacteria recovered from macrophages (blue symbols) exposed to 1000 × MIC RIF for 3 h. (F-G) NO and TNF-α were measured with commercial reagent kit. *S*. *a*, *S*. *aureus*. Data pooled from 2 independent experiments with 5 to 6 mice per group. ***p < 0.001, ****p < 0.0001 indicate significant differences from each group. Student’s t test analysis of variance was performed and the data are presented as the means ± SEM.

Recent studies revealed that the induction of bacterial persistence correlates significantly with host immune activation, in which reactive oxygen species and reactive nitrogen species may play a major role [21,22]. To further elucidate whether innate immune memory is involved in the development of antibiotic tolerance in recurrent *S*. *aureus* infections, we investigated the effects of prior infection on the peritoneal macrophages (PMs) and the intracellular *S*. *aureus* survival **(Fig 3A)**, which can be displayed on the laboratory growth plate as non-stable small colony variants (nsSCVs) due to extended lag periods before regrowth [23,24]. We showed that peritoneal macrophages obtained from *S*. *aureus*-primed mice had lower levels of intracellular bacteria, but most of which were tolerant to rifampicin (about 4.8 folds compared to the PBS controls), a bactericidal antibiotic commonly prescribed for intracellular *S*. *aureus* infections [25] **(Figs 3B, 3C and S1)**. Furthermore, we noticed a phenotypic adaptation as small-colony variants (SCVs) after 20 hours of bacterial plating, suggesting a switch to persister phenotype **(Fig 3D, left panel)**. The number of these nsSCVs was also significantly higher in PMs from *S*. *aureus*-primed mice than the PMs from non-primed mice. **(Fig 3D, right panel)**. Consistently, a regrowth and antibiotic sensitivity experiment showed all of the *S*. *aureus* cells could regrow and restore antibiotic sensitivity, as well as exhibiting a biphasic killing characteristic **(Fig 3E)**. The transient phenotypic switch indicated that these intracellular survivors are persisters [23]. Furthermore, the production of inflammatory mediator nitric oxide (NO) and cytokine TNF-α in prior *S*. *aureus* primed macrophages was higher than the unprimed macrophages upon lipopolysaccharide (LPS) restimulation after seven days of rest **(Fig 3F and 3G)**, suggesting previous *S*. *aureus* infection provoked trained immunity [26]. Collectively, these findings reveal that prior *S*. *aureus* infection triggered innate immune memory to protect against subsequent infection, whereas it contributed to increasing bacterial persistence.

### Trained immunity reduces the bacterial load in recurrent *S*. *aureus* skin infection but promotes the antibiotic persistence

**Fig 4:**
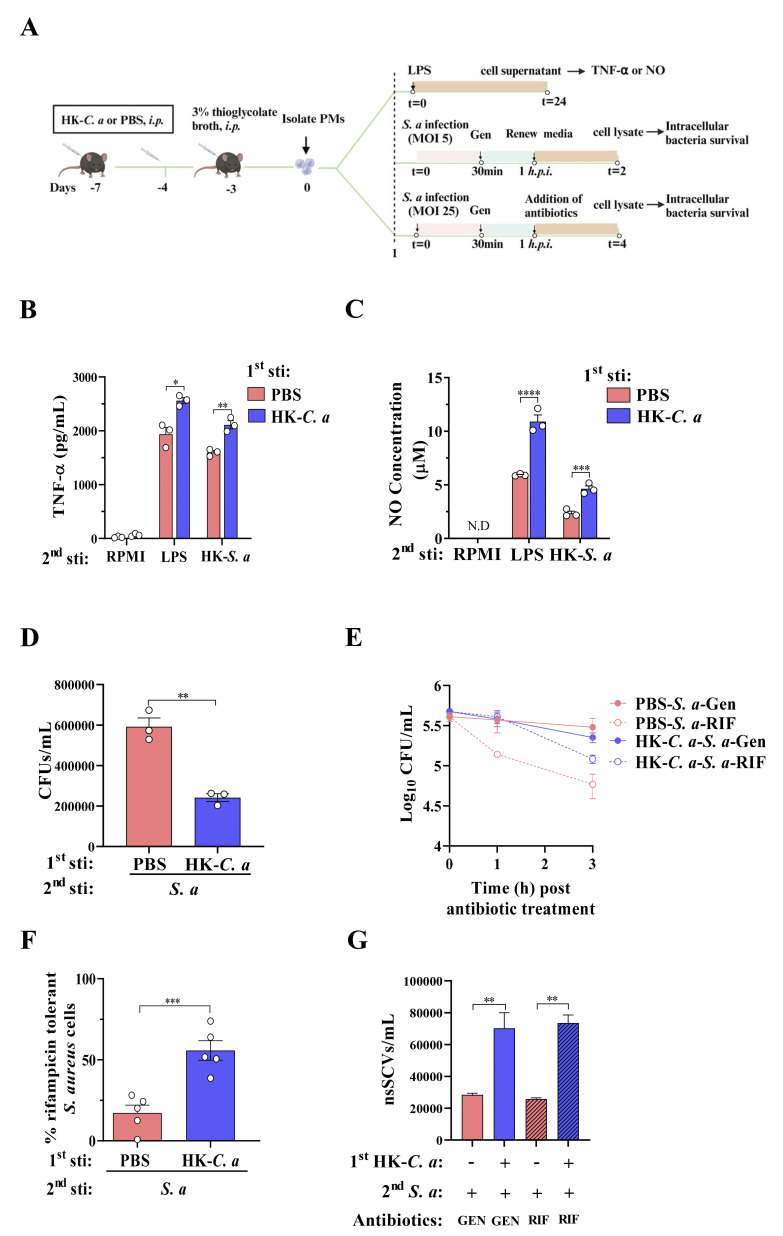
Trained immunity promotes antibiotic persistence of *S*. *aureus* in host macrophages. (A) Schematic representation. Mice were infected intraperitoneally with HK*-C*. *a* or PBS (i.p.) and 3 days later, 3% thioglycolate broth was injected. Four days later, the peritoneal macrophages were isolated and restimulated with LPS or Heat-killed *S*. *aureus* (HK-*S*. *a*), or reinfected with live *S*. *aureus* (*S*. *a*, MOI = 5 or 25). The cell samples were harvested and detected. Created by Biorender. (B-D) TNF-α (B), NO (C) and intracellular killing of *S*. *aureus* (D) in PMs from naïve (PBS) or HK-*C*. *a* trained mice, MOI = 5. (E) Survival of *S*. *aureus* cells in PMs from naïve (PBS) or HK-*C*. *a* trained mice, MOI = 25. (F) % Survival of *S*. *aureus* cells treated with rifampicin for 2 h compared to the gentamicin control (extrapolated from D). (G) The abundance of nsSCVs in PMs from naïve (PBS) or HK-*C*. *a* trained mice, MOI = 25. *p < 0.05, **p < 0.01, ***p < 0.001, ****p < 0.0001 indicate significant differences from each group. *S*. *a*, *S*. *aureus*. Data pooled from 2 independent experiments with 5 to 6 mice per group. Two-way ANOVA or student’s t test analysis of variance was performed and the data are presented as the means ± SEM.

To further determine whether antibiotic persistence is associated with trained immunity, we established an ex vivo experimental approach to mimic innate immune memory in mice **(Fig 4A)**. Consistent with our previous study [27], HK*-C*. *a*-trained PMs showed higher TNF-α and NO production, stronger bactericidal activity than naive PMs upon restimulation with LPS, heat-killed *S*. *aureus* (HK-*S*. *a*) or infected with live *S*. *aureus* seven days later **(Fig 4B–4D)**, suggesting that HK-*C*. *a* induced trained immunity in peritoneal macrophages. Meanwhile, the majority of intracellular *S*. *aureus* cells in naive PMs was susceptible to rifampicin, while the surviving bacteria in HK-*C*. *a* trained macrophages were markedly tolerant (about 3.3 folds compared to PBS treated macrophages) to rifampicin **(Fig 4E and 4F)**. The levels of nsSCVs in HK-*C*. *a*-trained macrophages were also significantly increased compared to naive macrophages **(Fig 4G)**. Furthermore, the tolerance and persistence of intracellular *S*. *aureus* cells against various antibiotics (i.e., ampicillin, kanamycin, tetracycline and vancomycin) in HK-*C*. *a* trained PMs were significantly higher than that of naïve PMs (**S2 Fig**). Similar phenomena were also observed in murine bone marrow-derived macrophages (**S3 Fig**). These results suggest that trained immunity attenuates intracellular *S*. *aureus* burden in macrophages but promotes the persisters formation.

**Fig 5:**
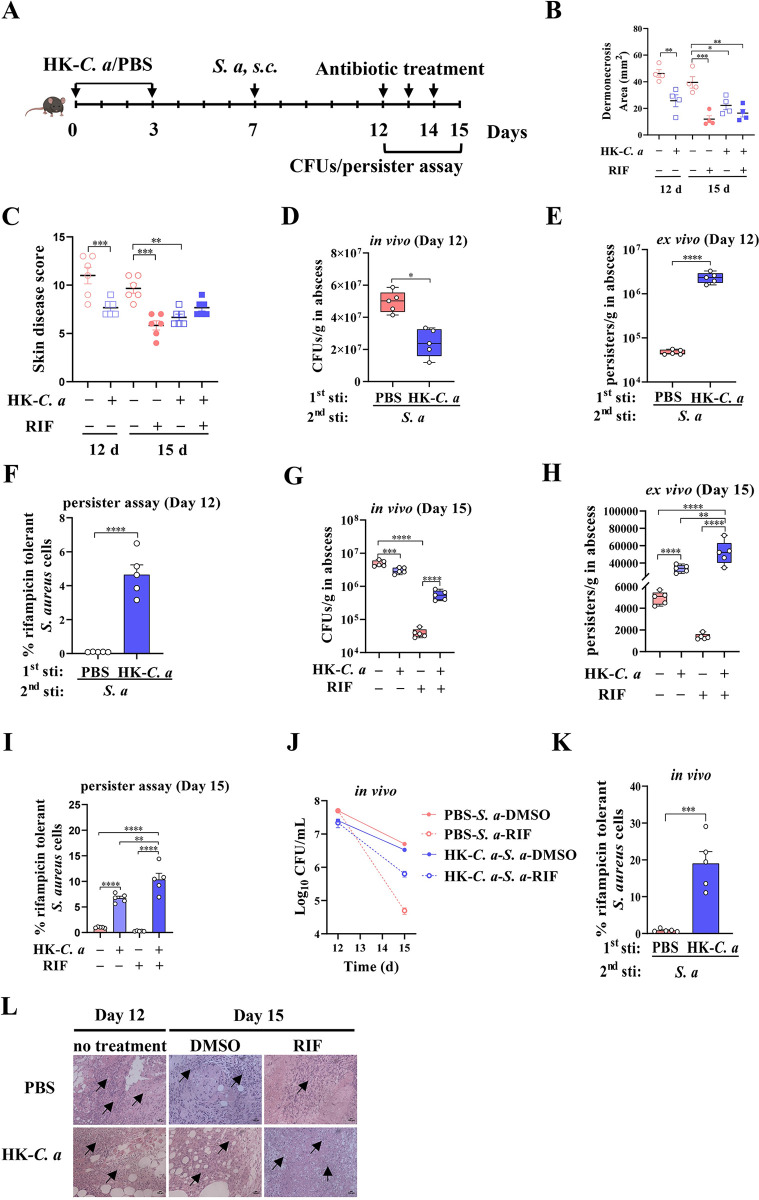
Trained immunity enhances antibiotic persistence of *S*. *aureus* in a murine SSTI model. (A) Scheme of the murine trained and infection model. Mice were infected intraperitoneally with HK-*C*. *a* or PBS (i.p.) on day 0 and day 3. On day 7, mice were subcutaneously infected with *S*. *aureus*. Five days post infection, they were treated with RIF for 3 days. Samples were collected before and after RIF treatment for detection. The diagram was originally hand-drawn by the first author. Skin lesion size (B) and skin disease score (C) of mice with different treatments at indicated time point after infection. (D) Bacterial load in abscess of mice at day 12. (E) Ex vivo persister assay of bacteria harvested from murine abscesses using RIF (8 μg/mL). Bacterial survival was assessed after 24 h. (F) Percentage of persister cells in murine abscess at day 12. (G-I) Bacterial load (G), persister assay (H), and percentage of persisters (I) in murine abscess at day 15. (J) Changes of bacterial load in murine abscess before and after RIF treatment. (K) % Survival of *S*. *aureus* cells treated with rifampicin for 3 d compared to the DMSO control (extrapolated from J). (L) H&E-stained skin sections (magnification of 100 ×). The black arrow indicates inflammatory cells infiltration. E: epidermis; D: dermis; A: adipose tissue; M: muscle fibres; *p < 0.05, **p < 0.01, ***p < 0.001, ****p < 0.0001 indicate significant differences from each group. *S*. *a*, *S*. *aureus*. Data pooled from 2 independent experiments with 5 to 6 mice per group. Student’s t test or one-way ANOVA analysis of variance was performed and the data are presented as the means ± SEM.

We next explored the effects of trained immunity on *S*. *aureus* persistence in recurrent SSTIs. To this end, mice were treated with HK-*C*. *a* twice intraperitoneally on day 0 and day 3, followed by subcutaneous *S*. *aureus* infection on day 7 and monitoring for 5 days until rifampicin treatment. **(Fig 5A)**. Our results showed that HK-*C*. *a* treated mice had reduced skin lesions size, skin disease score, *S*. *aureus* burden and pathological damage. **(Fig 5B–5D and 5L, left panel)**. Concomitantly, the bacteria obtained from abscesses were challenged with rifampicin for 4 h ex vivo to test for antibiotic persistence. We investigated a significant increase of the rifampicin persistence in HK-*C*. *a* trained mice than that of naïve mice **(Fig 5E and 5F)**, suggesting that trained immunity strongly promoted the increase of *S*. *aureus* persistence in recurrent SSTIs. In addition, HK-*C*. *a* trained mice showed more severe skin damages **(Fig 5B, 5C and 5L, right panel)** and higher levels of *S*. *aureus* burden compared to PBS treated mice **(Fig 5G)** after 3 days of antibiotic treatment, indicating that trained immunity attenuates the therapy effects of rifampicin. The number and frequency of the *S*. *aureus* persister cells in the HK-*C*. *a* trained mice with or without antibiotic treatment were significantly higher than that in naïve mice **(Fig 5H and 5I)**. The dynamic changes of *S*. *aureus* load before and after rifampicin treatment in mice showed a higher proportion of rifampicin-tolerance (about 22 folds) in HK-*C*. *a* trained mice compared to PBS treated mice **(Fig 5J and 5K)**. Overall, these results suggest trained immunity had an inducing effect on rifampicin persistence in subsequent *S*. *aureus* skin infection.

### The persistence of *S*. *aureus* induced by trained immunity may be related to the accumulation of fumarate

**Fig 6:**
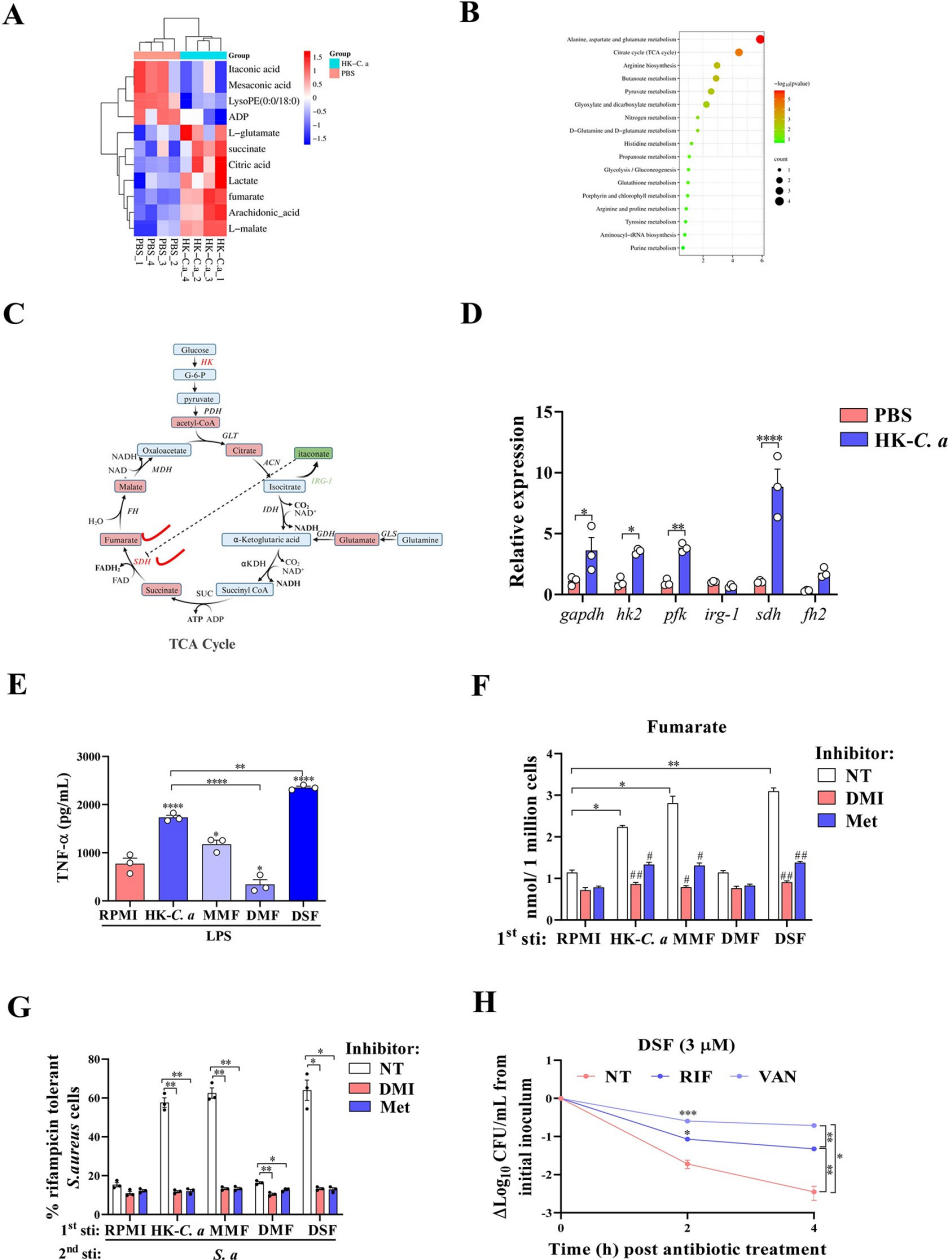
Trained immunity-mediated fumarate accumulation is associated with *S*. *aureus* persistence. PMs were isolated from mice and plated to flat-bottom plates. Cells were left to adhere for 24 h. Then, depending on the experiment inhibitors and/or HK-*C*. *a* were added. After 24 h incubation, cells were washed once with warm PBS and were left to rest in culture medium for five days (with one medium change half way). At day six, cells were restimulated with 50 ng/mL LPS for 24 h or obtained for metabolome analysis. (A) Cluster Heatmap of the major differential metabolites between HK-*C*. *a* and PBS group. Rows indicate different metabolites and columns indicate different conditions (n =  4) (B) KEGG analysis of the major differentially regulated metabolic pathways for the HK-*C*. *a*/ PBS group. (C) Schematic map of TCA cycle pathway depicting the major metabolic changes (filled rectangular box) in macrophages treated with HK-*C*. *a* versus PBS at day 6. Red, upregulated metabolites and genes. Green, downregulated metabolites and genes. Created by Biorender. (D) Quantitative real-time PCR analysis of partial glycolysis and citric acid cycle genes mRNA levels in PMs at day 6 before LPS restimulation. (E) Effects of initial stimulation with different fumarate derivatives on TNF-α production after LPS restimulation. (F) At day 6, cells were lysed and intracellular concentrations of fumarate were determined. (G) Effects of metformin and dimethyl itaconate on persister frequency of *S*. *aureus* cells in PMs treated with rifampicin for 2 h compared to the gentamycin control. (H) Time-killing curves against *S*. *aureus* in exponential phase culture preincubated with disodium fumarate (DSF) for 3 h and then exposed to 8 μg/mL RIF or 50 μg/mL vancomycin (VAN) for the indicated periods. *S*. *a*, *S*. *aureus*. Data pooled from 2 independent experiments with 3 replicates per group. *p < 0.05, **p < 0.01, ***p < 0.001, ****p < 0.0001; #p < 0.05, ##p < 0.01 indicate significant differences from each group. Student’s t test, one-way ANOVA or two-way ANOVA analysis of variance was performed and the data are presented as the means ± SEM.

The metabolic hallmarks of trained immunity are increased aerobic glycolysis and tricarboxylic acid (TCA) cycle [18]. To explore the intrinsic mechanism of trained immunity promoting *S*. *aureus* persistence, we performed intracellular metabolome analysis of macrophages stimulated with HK-*C*. *a* or PBS and examined the expression of some genes related to glycolysis and the TCA cycle. Metabolomics analysis indicated that the main differential metabolites were enriched in Citrate cycle (TCA cycle) and the alanine, aspartate, and glutamate metabolism pathways **(Figs 6A–6C and S4)**. HK-*C*. *a* stimulation also led to an increase in mRNA levels for *gapdh*, *hk2*, *pfk*, *sdh* and *fh2* genes, with the exception of *irg-1* **(Fig 6D)**. This gene encodes the enzyme immune-responsive gene 1 (IRG1), which is responsible for decarboxylating the TCA cycle intermediate cis-aconitate to itaconate [28]. Importantly, the mRNA level of succinate dehydrogenase (SDH), which oxidises succinate to fumarate, was increased significantly after HK-*C*. *a* stimulation. This is consistent with previous studies that trained immunity promotes the imbalance of SDH/IRG-1 axis in TCA cycle [29]. Recent studies revealed that monomethyl fumarate (MMF) could induce trained immunity phenotypes [30], but dimethyl fumarate (DMF) inhibited the increased aerobic glycolysis in inflammatory immune-cell subsets [31]. Previous studies have also found that disodium fumarate (DSF) can promote persistence of *Escherichia coli* against antibiotics [32]. These studies suggest that fumarate derivatives exhibite differentiated biological functions. We first investigated the potential role of these fumarate derivatives in trained immunity. Stimulation of macrophages with MMF and DSF on day 0 induced increased TNF-α production upon restimulation on day 6, whereas DMF did not induce this effect **(Fig 6E)**. This is consistent with the increase of intracellular fumarate induced by MMF and DSF, but not DMF **(Fig 6F)**. Inhibition of SDH or mTOR by dimethyl itaconate (DMI) or metformin respectively, also decreased TNF-α expression and fumarate concentrations in trained macrophages **(Figs 6F, S5 and S6)**. In addition, we observed the increased intracellular *S*. *aureus* persisters in macrophages trained with MMF and DSF, which were counteracted by DMI and metformin **(Fig 6G)**. These data suggest that fumarate is a crucial link between trained immunity and the *S*. *aureus* persistence.

To further investigate whether fumarate directly induces *S*. *aureus* persistence, we pretreated *S*. *aureus* with these fumarate derivates and tested the bactericidal effect of antibiotics. The results demonstrated that DSF could significantly increase the persistence of *S*. *aureus* to rifampicin and vancomycin, but not MMF or DMF **(Figs 6H, S7A–S7D and S8A)**. Of note, metformin significantly reduced the *S*. *aureus* persistence to rifampicin, whereas DMI did not exhibit this effect **(S7E, S7F, S8B and S8C Figs)**. Similarly, metformin also reduced the *S*. *aureus* persistence induced by DSF to rifampicin and vancomycin **(S8D and S8E Fig)**. Collectively, these results suggest that the accumulation of fumarate triggered by trained immunity has the potential to enhance the formation of *S*. *aureus* persisters, which provides a new promising adjuvant therapeutic target for recurrent *S*. *aureus* infections.

### A combination therapy of metformin/rifampicin attenuates bacterial burden and persistence in recurrent *S*. *aureus* SSTI

**Fig 7:**
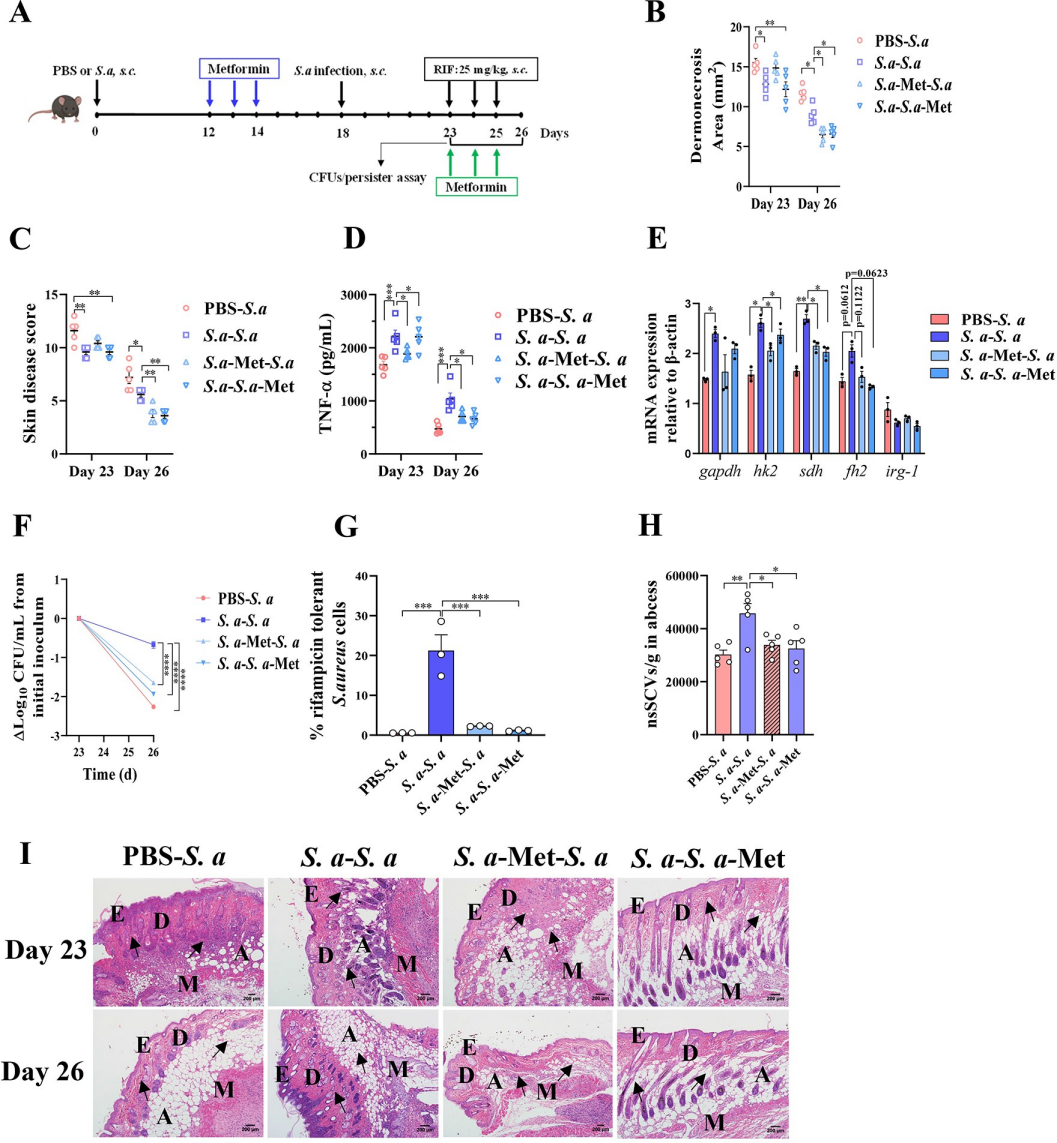
Metformin/rifampicin combination decreases *S*. *aureus* load and persisters in Mice. (A) Scheme of the murine skin infection model. The diagram was originally hand-drawn by the first author. Skin lesion size (B), skin disease score (C), TNF-α (D), the relative mRNA expression of *gapdh*, *hk2*, *sdh*, *fh2* and *irg-1* (E), numbers of *S*. *aureus* CFU (F) in the skin was measured at indicated time point. (G) % Survival of *S*. *aureus* cells treated with rifampicin for 3 d compared to the DMSO control. (H) The abundance of nsSCVs in the skin of mice with different treatments. (I) H&E-stained skin sections showed severe inflammatory injury after *S*. *aureus* infection (magnification of 100 ×). The black arrow indicates inflammatory cells infiltration. E: epidermis; D: dermis; A: adipose tissue; M: muscle fibres. *S*. *a*, *S*. *aureus*. Data pooled from 3 independent experiments with 5 to 6 mice per group. *p < 0.05, **p < 0.01, ***p < 0.001, ****p < 0.0001 indicate significant differences from each group. One-way ANOVA or two-way ANOVA analysis of variance was performed and the data are presented as the means ± SEM.

Since the ability of metformin to potently suppress trained immunity has been well documented [33,34], we sought to assess the efficacy of the metformin/antibiotic combination in a recurrent *S*. *aureus* infection model. Mice were infected with *S*. *aureus* 18 days before reinfection with an identical dose, followed by rifampicin treatment for 3 days at five days post-reinfection **(Fig 7A)**. Metformin was administered daily for 3 days at two time points (before reinfection or after reinfection). We verified previous *S*. *aureus* infection led to colonization protection, as indicated by the presence of smaller skin lesions and lower skin disease score throughout the infection period compared with PBS controls, consistent with previous findings **(Fig 7B and 7C)** [17,35]. Furthermore, the upregulation of inflammatory factor TNF-α and partial key metabolic genes *sdh*, *gapdh*, *hk2* in mice with prior *S*. *aureus* infection reflected the trained immunity signatures **(Fig 7D and 7E)**. Both methods of metformin and rifampicin combined treatment reduced skin lesions size, skin disease score, TNF-α expression and pathological damage to a greater extent than rifampicin therapy alone by day 26 in a recurrent skin infection **(Fig 7B–7D and 7I)**. The mRNA expression of hexokinase gene *hk2* and succinate dehydrogenase gene *sdh* was also downregulated by metformin therapy. Importantly, the combination therapy markedly enhanced rifampicin bactericidal activity in a recurrent *S*. *aureus* skin infection **(Fig 7F)**. In line with total bacterial burden, the number of rifampicin-tolerant bacteria and nsSCVs was also significantly reduced by combination therapy (90% and 95% for RIF tolerant *S*. *aureus*, 74% and 71% for nsSCVs) compared with rifampicin alone therapy in mice with a recurrent *S*. *aureus* skin infection **(Fig 7G and 7H)**. Overall, these results demonstrate that metformin has a synergistic effect on rifampicin therapy for recurrent *S*. *aureus* infections, especially when it comes to the bacterial persisters. Thus, combination of trained immunity inhibitors with antibiotic therapy may provide a novel strategy for recurrent *S*. *aureus* skin infections in the future.

## Discussion

Persister cells, a subpopulation of growth-arrested cells that display transient tolerance to lethal doses of antibiotics, are commonly associated with chronic or recurrent infections [36,37]. *S*. *aureus* SSTIs are associated with frequent recurrence. In this study, we conducted a prevalence survey of *S*. *aureus* persisters in bovine mastitis and showed that persister frequency is significantly associated with previous infection history. This is similar to a recent study that *E*. *coli* tolerance in bloodstream infections correlates with past episodes [15]. Our study also revealed previous *S*. *aureus* infection led to *S*. *aureus* persistence to killing by rifampicin in subsequent infection in vivo and in vitro. Previous uropathogenic *Escherichia coli* infection induces trained immune responses in bladder epithelial stem cells that affect susceptibility to recurrent infection [38]. In addition, the recurrent *S*. *aureus* skin infection has shown that macrophage-conferred trained immunity signatures [17], but the relationship between trained immunity and bacterial persistence is poorly understood. Using a HK-*C*. *a* induced trained immunity model in vivo and in vitro, we showed that activation of trained immunity contributed to rifampicin persistence of *S*. *aureus* in secondary infection, where it reduced the effectiveness of antibiotic treatment and increased disease severity.

*S*. *aureus* persisters switch to nonstable small colony variants (nsSCVs) by transcriptomic reprogramming and remain metabolically active [23,39]. Here, we found a broad heterogeneity in colony radius of intracellular *S*. *aureus* at 24 h for plated immediately after antibiotic treatment. Once the antibiotic pressure is removed, the persisters regrow and give rise to a susceptible population [40]. Indeed, we observed a full reversion of the phenotype in terms of both growth resumption and susceptibility towards rifampicin of intracellular *S*. *aureus* from trained macrophages after reinoculation in broth. These results evidenced that these intracellular *S*. *aureus* subpopulations are persisters.

Trained immunity promoted the expression of succinate dehydrogenase (SDH), leading to the accumulation of intracellular fumarate, which further accelerates the training effect [29,41]. Here, we also observed a significant increase in the expression of SDH and the accumulation of fumarate in macrophages after training. Sodium fumarate, as a fumarate derivative, has been reported to induce antibiotic persistence in *Escherichia coli*, partly due to its terminal electron acceptor function [32]. In this study, we found that sodium fumarate induced the persistence of *S*. *aureus* to vancomycin and rifampicin in vitro, the two commonly used antibiotics in MRSA infections. However, other fumarate derivatives, including monomethyl fumarate and dimethyl fumarate, did not induce antibiotic persistence, despite their significant immunomodulatory properties. This is in line with some studies that dimethyl fumarate can be employed as an antibiotic adjuvant to effectively treat *S*. *aureus* persistent infections, due to the antagonistic effect on GAPDH and downregulation of glycolysis [31,42,43]. Whereas, monomethyl fumarate has been reported to have the function of driving innate immunity and inducing trained immunity [30,44]. Intriguingly, we investigated that sodium fumarate also induced trained immunity. Therefore, we demonstrated that the accumulation of fumarate induced by trained immunity, particularly sodium fumarate, is a significant host factor in the formation of *S*. *aureus* persisters.

The accumulation of fumarate in macrophages may induce the formation of persisters in several ways. First, exposure of *S*. *aureus* to fumarate resulted in higher intracellular fumarate accumulations in *S*. *aureus* [32], which might trigger a ppGpp-dependent mechanism due to carbon source transition [45]. Because the intracellular environment of macrophages lacks nutrients (e. g. glucose, amino acid etc.) for *S*. *aureus* survival, the intracellularly accumulated fumarate might be used as a second carbon source. This carbon source transition leads to a ppGpp-dependent stringent response for intracellular Staphylococcus aureus. Second, the accumulation of fumarate in *S*. *aureus* cells may affect the electron transport chain, resulting in a decrease in proton motive force (PMF). PMF is the driving force for ATP synthesis [46], which is critical for the functions of efflux pump [47]. The decreased ATP levels in *S*. *aureus* cells promote formation of endogenous protein aggregates, impair the efflux of antibiotics, and increase antibiotic persisters and dormancy [48]. Third, intracellular fumarate in *S*. *aureus* cells might reduce the generation of hydroxyl radicals (·OH). The production of hydroxyl radicals has been suspected to be a common mechanism of antibiotic-induced cell death [49–51]. Traditionally, ·OH is generated during electron transport to oxygen [29]. However, fumarate-mediated persisters and *hipA7* mutant displayed reduced ·OH formation even though the cells were grown under aerobic conditions [32]. Therefore, persisters may also avoid ·OH production by detouring electrons from ETC to fumarate, just as aerobic glycolysis.

Because the drug prevention of *S*. *aureus* infection is impractical, it is very important for the treatment after infection. Metformin, as a known inhibitor of trained immunity [34], we demonstrate it reduces the accumulation of fumarate in macrophages. In vitro, metformin also promotes the sensitivity of *S*. *aureus* to antibiotics and attenuates the antibiotic persistence induced by sodium fumarate. However, its underlying mechanism needs further research. Additionally, the combination of metformin and antibiotics significantly reduced the severity of skin infections caused by *S*. *aureus*, indicating that metformin can serve as a potential antibiotic adjuvant to combat bacterial infections.

In conclusion, our study reveals previous *S*. *aureus* infection can induce innate immune memory to protect against secondary infection and promote *S*. *aureus* persistence through the accumulation of fumarate in macrophages, which providing compelling arguments for drug screening strategies that incorporate the host environment. Furthermore, as an inhibitor of trained immunity, metformin has been shown to reverse the trained innate immune memory phenotype and reduce the frequency of *S*. *aureus* persisters. Finally, there is an intriguing prediction implied by this work: the identification of compounds that inhibit trained immunity and reverse an antibiotic-tolerant phenotype could represent a novel type of drug that would show broad spectrum synergy when used in combination with existing frontline antibiotics.

## Materials and methods

### Ethical statement

All animal studies were subject to approval by the Institutional Animal Care and Use Committee (IACUC) of Jilin University (China). The full proposal was considered by the IACUC ethics committee, which approved the animal care and use permit license (KT201902111). All experiments comply with the manual of the care and use of laboratory animals published by the US National Institutes of Health.

### Materials

Methyl-fumarate (MMF), dimethyl fumarate (DMF), disodium fumarate (DSF), dimethyl itaconate (DMI) and rifampicin were purchased from Macklin (Shanghai, China). Enzyme-linked immunosorbent assay (ELISA) kit for TNF-α were bought from SinoBiological (Beijing, China). The nitric oxide (NO) detection kit was purchased from Beyotime (Nanjing, China). The fumarate detection kit was purchased from Jingmei Biotechnology (Yancheng, China).

### Bacterial growth conditions

For exponential growth conditions, bacteria were grown in tryptic soy broth (TSB) overnight (16 h), diluted, and regrown in TSB for 2 h.

### Tolerant disk test

The Tolerance Disk (TD) test was performed as previously described with some modifications [15]: bacterial cultures grown overnight were adjusted to an optical density of 0.1 at 600 nm (OD600). 100 μl of the bacterial suspension was plated evenly on Mueller Hinton agar plates and 10 μl of rifampicin solution was pipetted onto blank antimicrobial susceptibility Oxoid disks. The final amount of rifampicin on the disks was 0.05 μg, 0.5 μg, and 5 μg and plates were incubated overnight at 37°C for 18 h (step I). In step II, 10 μl of a solution containing 40% glucose and 20% casamino acids mixture was added to the antibiotic disk and the plates were reincubated at 37°C for another 18 h. If more than 20 microcolonies appeared within the antibacterial zone after step II, Strains were defined as’ tolerant ’.

### Trained immunity model

C57BL/6J mice were injected intraperitoneally with 100 μl HK-*C*. *a* (1×10^6^ CFU) or PBS on -7 and -4 days respectively. Peritoneal macrophages were harvested from mice by peritoneal lavage 3 days after the i.p. injection with 4 mL of 3% thioglycolate on -3 days. PMs were seeded in 96-well plates in RPMI 1640 (Gibco) supplemented with 10% fetal bovine serum (FBS) and antibiotics (Penicillin-Streptomycin; Gibco) overnight in a humidified incubator at 37°C/5% CO_2_. Next day, cells were re-stimulated with LPS (100 ng/mL; *E*. *coli* O55:B5; Sigma-Aldrich), HK-*S*. *a* (MOI 5) in RPMI 1640 for 24 hours. Supernatant was harvested for quantification of TNF-α and nitric oxide release.

### Experiments in broth

For time-kill curves, *S*. *aureus* was grown overnight in TSB broth, diluted in fresh medium to reach a starting OD600 nm of 0.01 and grown to the mid-exponential phase. Cultures were washed in PBS twice, re-suspended in fresh TSB medium and diluted to a starting inoculum of 5 × 10^8^ CFU/mL and exposed to rifampicin at different multiples of MIC, for the indicated times. For CFU counting, samples were diluted in PBS before plating on Tryptic Soy agar. Data are expressed as log10 CFU per mL lysates.

### Intracellular persistence model

Infection was performed following a protocol adapted from Peyrusson F et al [40]. Briefly, primed or non-primed PMs (2 × 10^5^) were seeded into 24-well plate in 0.5 mL of medium and infected with *S*. *aureus* USA300 at a multiplicity of infection of 25 for 30 min to allow phagocytosis. Cells were then washed with PBS, and non-phagocytized bacteria were eliminated by a 30 min incubation at 37°C in RPMI 1640 supplemented with 100 μg/mL gentamicin (Sigma). Gentamicin was eliminated by washing in PBS, after which cells were reincubated at 37°C in RPMI 1640 with 10% FBS in the presence either of 10 μg/mL of gentamicin (to prevent extracellular growth; control conditions), or rifampicin at 1000 × MIC for the indicated times. Cells were then washed with PBS and lysed with PBS containing 0.1% (w/v) Triton X-100 (Sigma) to release intracellular bacteria. Lysates were serial-fold diluted in PBS before plating on Tryptic Soy agar. CFU were enumerated and the colony phenotype determined. Data are expressed as log10 CFU per mL lysates.

### Murine infection model

Primed or non-primed C57BL/6J mice were injected into the flanks with *S*. *aureus* to induce abscess formation as previously described [1]. Mice were killed either 5 or 8 d after infection depending on the treatment regimen. Skins were assessed in a blinded fashion using a scoring system described previously [52]. Skin samples were fixed in 10% formalin and processed for H&E. Persister levels were determined by performing a persister assay with RIF.

### Persister assay

Approximately 1 × 10^7^ CFU/mL bacteria were inoculated in TSB broth supplemented with rifampicin (1000 × MIC). After 4 h, bacterial survival was determined by CFU enumeration and calculated relative to the inoculum. Exponential phase bacteria were used to test for baseline survival levels.

### NO production

The generation of nitric oxide (NO) was measured by the Griess reaction. The NO production from culture supernatants was measured according to the indication on the NO assay kit (Beyotime S0021S).

### Metabolome analysis

Peritoneal macrophages were seeded in 90mm-cell culture dishes, three dishes for each condition. After washing with warm PBS, PMs were incubated with culture medium only as a negative control or 1% volume of HK-*C*. *a* (OD_600 nm_ = 1) for 24 h (in 10% pooled fetal bovine serum). Cells were washed once with 2 mL warm PBS and incubated for 5 days in culture medium with 10% serum and medium was changed once. Cells were trypsinized and live cells were counted. The cell pellets (about 10^7 cells) were snap frozen and stored at −80°C until metabolite analysis. Metabolites were extracted in the extraction solution (MeOH:CAN:H2O, 2:2:1 (v/v)), and profiled by liquid chromatograph coupled to tandem mass-spectrometry(LC-MS/MS). LC-MS/MS analyses were performed using an UHPLC system (Vanquish, Thermo Fisher Scientific) with a Waters ACQUITY UPLC BEH Amide (2.1 mm × 50 mm, 1.7 μm) coupled to Orbitrap Exploris 120 mass spectrometer (Orbitrap MS, Thermo). The mobile phase consisted of 25 mmol/L ammonium acetate and 25 ammonia hydroxide in water(pH = 9.75)(A) and acetonitrile (B). The auto-sampler temperature was 4°C, and the injection volume was 2 μL. The Orbitrap Exploris 120 mass spectrometer was used for its ability to acquire MS/MS spectra on information-dependent acquisition (IDA) mode in the control of the acquisition software (Xcalibur, Thermo). In this mode, the acquisition software continuously evaluates the full scan MS spectrum. The ESI source conditions were set as following: sheath gas flow rate as 50Arb, Aux gas flow rate as 15 Arb, capillary temperature 320°C, full MS resolution as 60000, MS/MS resolution as 15000, collision energy: SNCE 20/30/40, spray voltage as 3.8 kV (positive) or -3.4 kV (negative), respectively.

The raw data were converted to the mzXML format using ProteoWizard and processed with an in-house program. which was developed using R and based on XCMS, for peak detection, extraction, alignment, and integration. The R package and the BiotreeDB(V3.0)were applied in metabolite identification [53].

### RT-PCR

RNA was isolated from cultured cells using the TRIzol reagent (Takara) and reverse transcribed to cDNA using High-Capacity Reverse Transcription Kit (Applied Biosystems, Bleiswijk, The Netherlands). RT-PCR was performed on a Quantstudio 3. Real-Time PCR System using predesigned prime sets (Applied Biosystems). The housekeeping gene *β-actin* was used to normalize CT values for each sample. Relative mRNA quantity was calculated using the 2^-ΔΔCt^ (fold change) method as indicated in the figure legends.

### Statistical analysis

Graphpad Prism 8.3 (Graphpad Software, La Jolla, CA, USA) was used for graph design and statistical analyses. Depending on the data distribution, independent-samples Student’s t-test, one-way ANOVA or two-way ANOVA followed by Bonferroni post hoc analysis was used to compare two or more groups. *P < 0.05, **P < 0.01, ***P < 0.001, ****P < 0.0001.

### Ethics approval

All animal studies were subject to approval by the Institutional Animal Care and Use Committee (IACUC) of Jilin University (China). The full proposal was considered by the IACUC ethics committee, which approved the animal care and use permit license (KT201902111). All experiments comply with the manual of the care and use of laboratory animals published by the US National Institutes of Health.

## Supporting information

S1 Fig Time-kill curves against *S*. *aureus* in exponential phase culture exposed to different concentrations of rifampicin or not for the indicated periods. (TIF)

S2 Fig Persistence of *Staphylococcus aureus* in peritoneal macrophages to different antibiotics. (A) Determination of intracellular non-stable small colony variants (nsSCVs) in mouse peritoneal macrophages. (B) The antibiotic tolerance frequency of *S*. *aureus* in PMs from naïve (PBS) and HK-*C*. *a* trained mice to different antibiotics. VAN, vancomycin. AMP, Ampicillin. KAN, kanamycin. TET, tetracycline. *S*. *a*, *S*. *aureus*. Data pooled from 3 independent experiments with 3 replicates per group. *p < 0.05, **p < 0.01, ***p < 0.001, ****p < 0.0001 indicate significant differences from each group. Two-way ANOVA analysis of variance was performed and the data are presented as the means ± SEM.
(TIF)

S3 Fig Persistence of *Staphylococcus aureus* in murine bone marrow-derived macrophages (mBMDM) to rifampicin treatment. (A) Determination of intracellular non-stable small colony variants in mBMDM. (B) The rifampicin tolerance frequency of *S*. *aureus* in mBMDM pretreated with PBS or HK-*C*. *a*. *S*. *a*, *S*. *aureus*. Data pooled from 3 independent experiments with 3 replicates per group. **p < 0.01, ***p < 0.001 indicate significant differences from each group. One-way ANOVA and student’s t analysis of variance was performed and the data are presented as the means ± SEM.
(TIF)

S4 Fig Metabolome analysis of macrophages stimulated with HK-*C*. *a* by untargeted metabolome profiling. (A) Principal component analysis (PCA) score plot and (B) volcano plot of differential metabolites (p < 0.05) between PBS and HK-*C*. *a-*treated macrophages. (C-H) Box plot of the differential metabolites correlated with TCA cycle for PBS vs HK-*C*. *a-*treated macrophages. n = 4 biological replicates per group. *p < 0.05, **p < 0.01 indicate significant differences from each group. Student t test analysis of variance was performed and the data are presented as the means ± SEM.
(TIF)

S5 Fig The effects of DMI and Met on trained immunity. (A-B) Mouse peritoneal macrophages were trained with HK-*C*. *a* or left in culture medium for 24 h in the presence or absence of SDH inhibitor (DMI) or mTOR inhibitor (Met). After 5 days, cells were restimulated with LPS to determine NO (A) and TNF-α (B) production. The data are shown as means ± SEM, Data pooled from 3 independent experiments with 3 replicates per group. Significance was determined using a one-way ANOVA with a Dunnett’s multiple comparisons test; *p < 0.05, ***p < 0.001, ****p < 0.0001.
(TIF)

S6 Fig In vitro cytotoxicity experiment using CCK8 assay. Peritoneal macrophages were treated with MMF (A), DMF (B), DSF (C), DMI (D), and Met (E) at different concentration conditions. Each data is presented as the means ± SEM. Significance was determined using a one-way ANOVA with a Dunnett’s multiple comparisons test; *p < 0.05, **p < 0.01, ***p < 0.001, ****p < 0.0001 vs Control.
(TIF)

S7 Fig The cytotoxicity of fumarate derivatives, DMI and metformin on *S*. *aureus*. (A) Structural formula of fumaric acid and its derivatives. (B-F) The growth curves of *S*. *aureus* in exponential phase culture co-incubated with different concentrations of MMF (B), DMF (C), DSF (D), Met (E), and DMI (F).
(TIF)

S8 Fig Effects of pre-exposure to fumarate derivatives or trained immunity inhibitors on *S*. *aureus* persistence levels. (A) Time-kill curves of *S*. *aureus* pre-incubated with MMF, DMF, and DSF followed by rifampicin treatment. (B-C) Fraction of surviving cells of *S*. *aureus* pre-treated with DMI (B) or Met (C) after secondary treatment with rifampicin or vancomycin for indicated times. (D-E) Fraction of surviving cells of *S*. *aureus* pre-treated with DSF and Met after secondary treatment with rifampicin (D) or vancomycin (E) for indicated times. Significance was determined using a two-way ANOVA with a Tukey multiple comparisons test; *p < 0.05, ***p < 0.001, ****p < 0.0001.
(TIF)

S1 Table Baseline characteristics of mastitis cows with *S*. *aureus* infections. (DOCX)

S1 Data Excel spreadsheet containing, in separate sheets, the data points presented in Figs 1–7 and S1–S8. (XLSX)
