# Supplementary figures and images for: Trained immunity in recurrent Staphylococcus aureus infection promotes bacterial persistence
Source: PLoS Pathog. 2024 Jan 19;20(1):e1011918. doi: 10.1371/journal.ppat.1011918 (PMC10798626; doi:10.1371/journal.ppat.1011918)

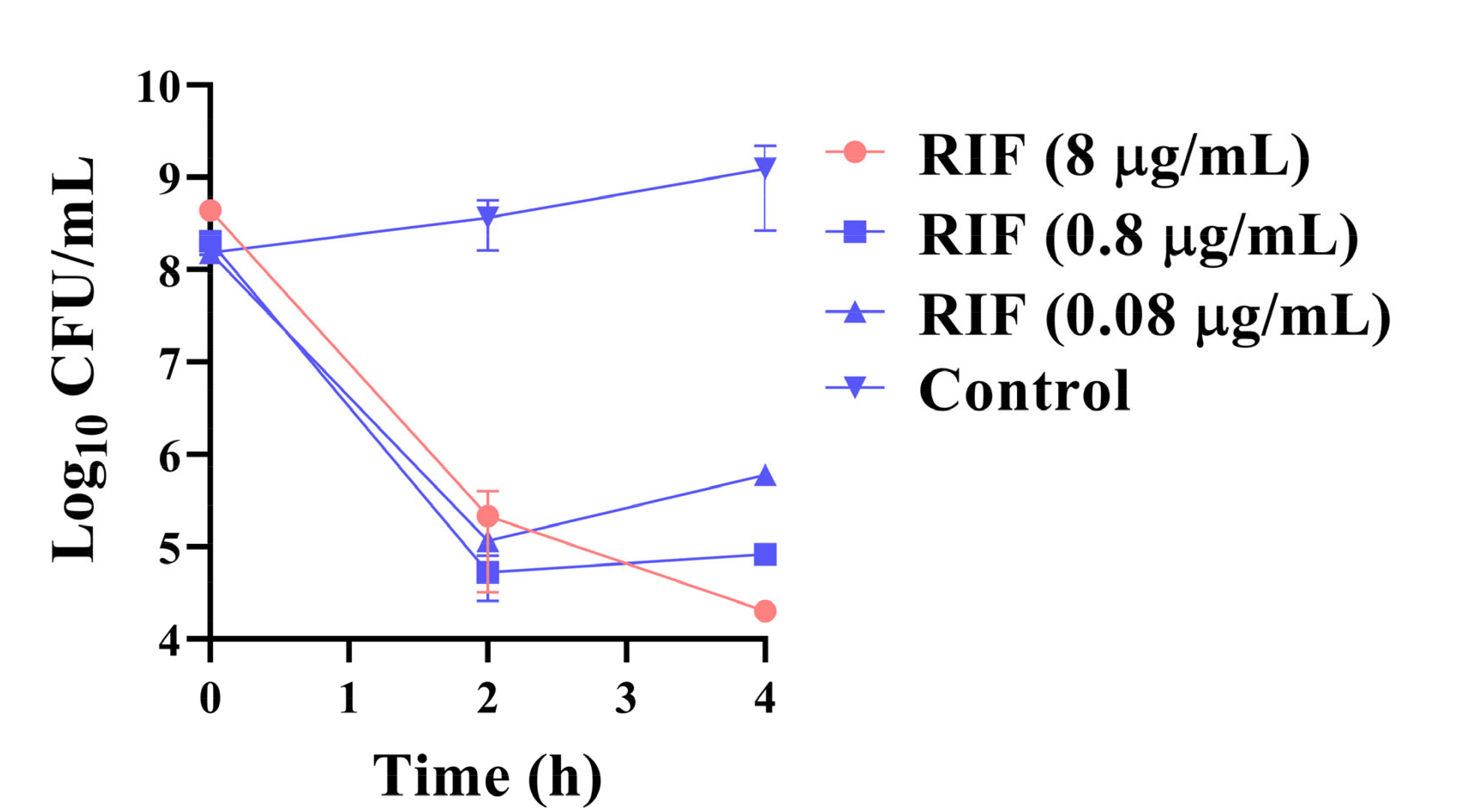

Supplement: S1 Fig — (TIF) [file ppat.1011918.s001.tif]

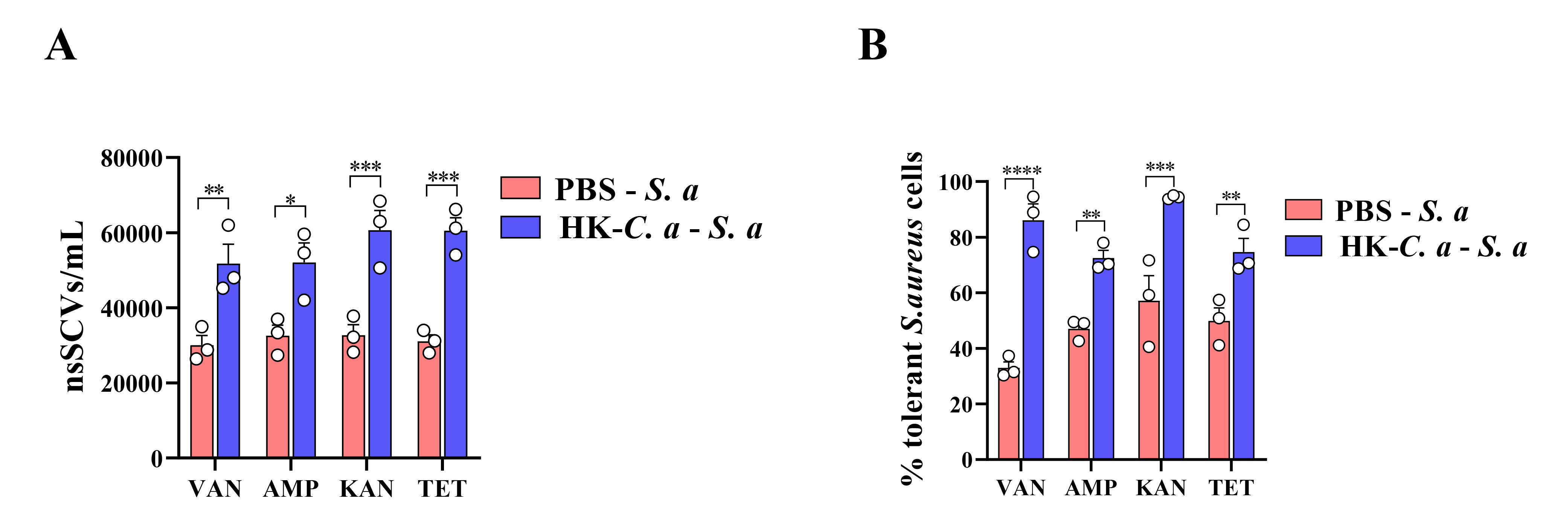

Supplement: S2 Fig — (A) Determination of intracellular non-stable small colony variants (nsSCVs) in mouse peritoneal macrophages. (B) The antibiotic tolerance frequency of S. aureus in PMs from naïve (PBS) and HK-C. a trained mice to different antibiotics. VAN, vancomycin. AMP, Ampicillin. KAN, kanamycin. TET, tetracycline. S. a, S. aureus. Data pooled from 3 independent experiments with 3 replicates per group. *p < 0.05, **p < 0.01, ***p < 0.001, ****p < 0.0001 indicate significant differences from each group. Two-way ANOVA analysis of variance was performed and the data are presented as the means ± SEM. (TIF) [file ppat.1011918.s002.tif]

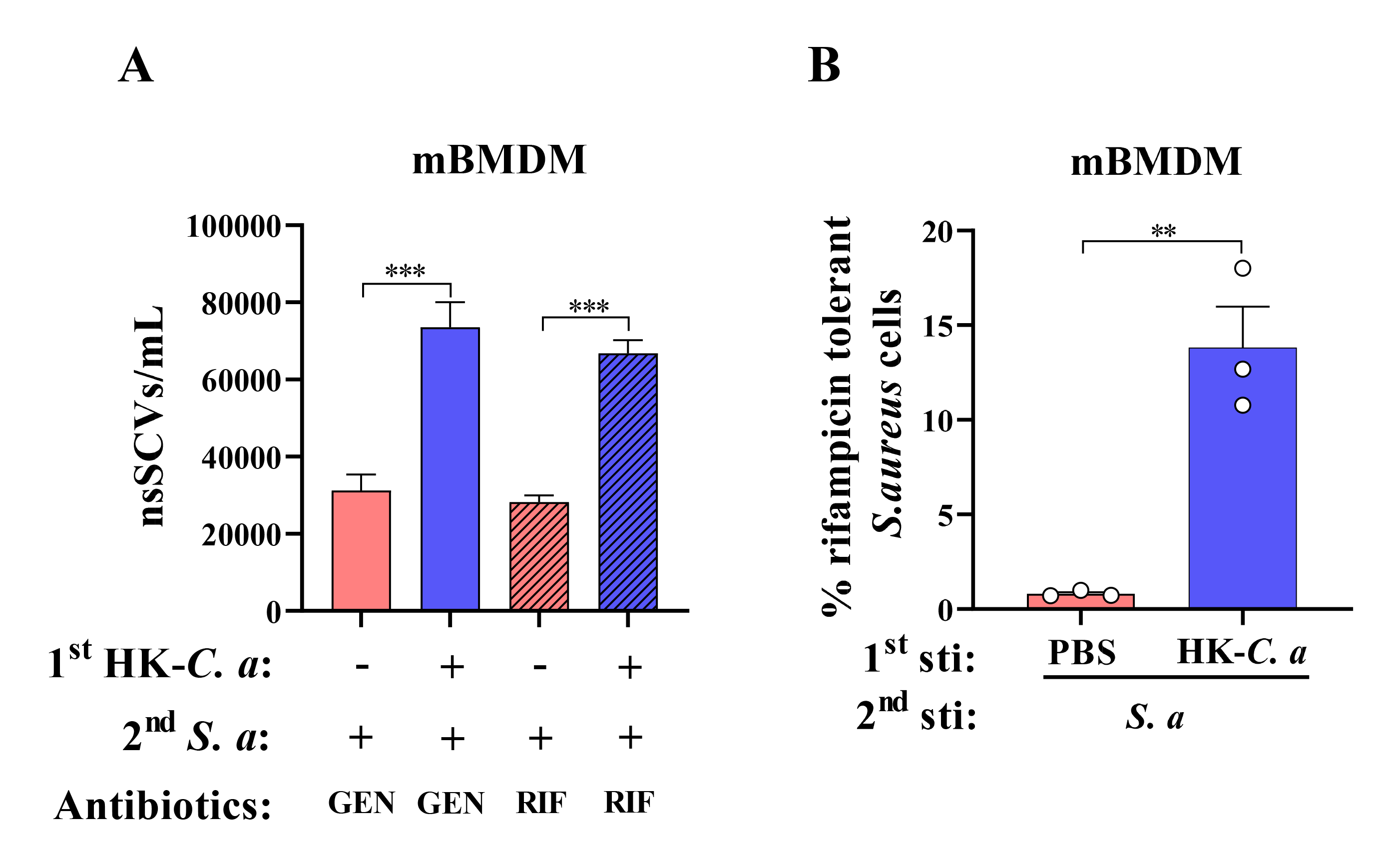

Supplement: S3 Fig — (A) Determination of intracellular non-stable small colony variants in mBMDM. (B) The rifampicin tolerance frequency of S. aureus in mBMDM pretreated with PBS or HK-C. a. S. a, S. aureus. Data pooled from 3 independent experiments with 3 replicates per group. **p < 0.01, ***p < 0.001 indicate significant differences from each group. One-way ANOVA and student’s t analysis of variance was performed and the data are presented as the means ± SEM. (TIF) [file ppat.1011918.s003.tif]

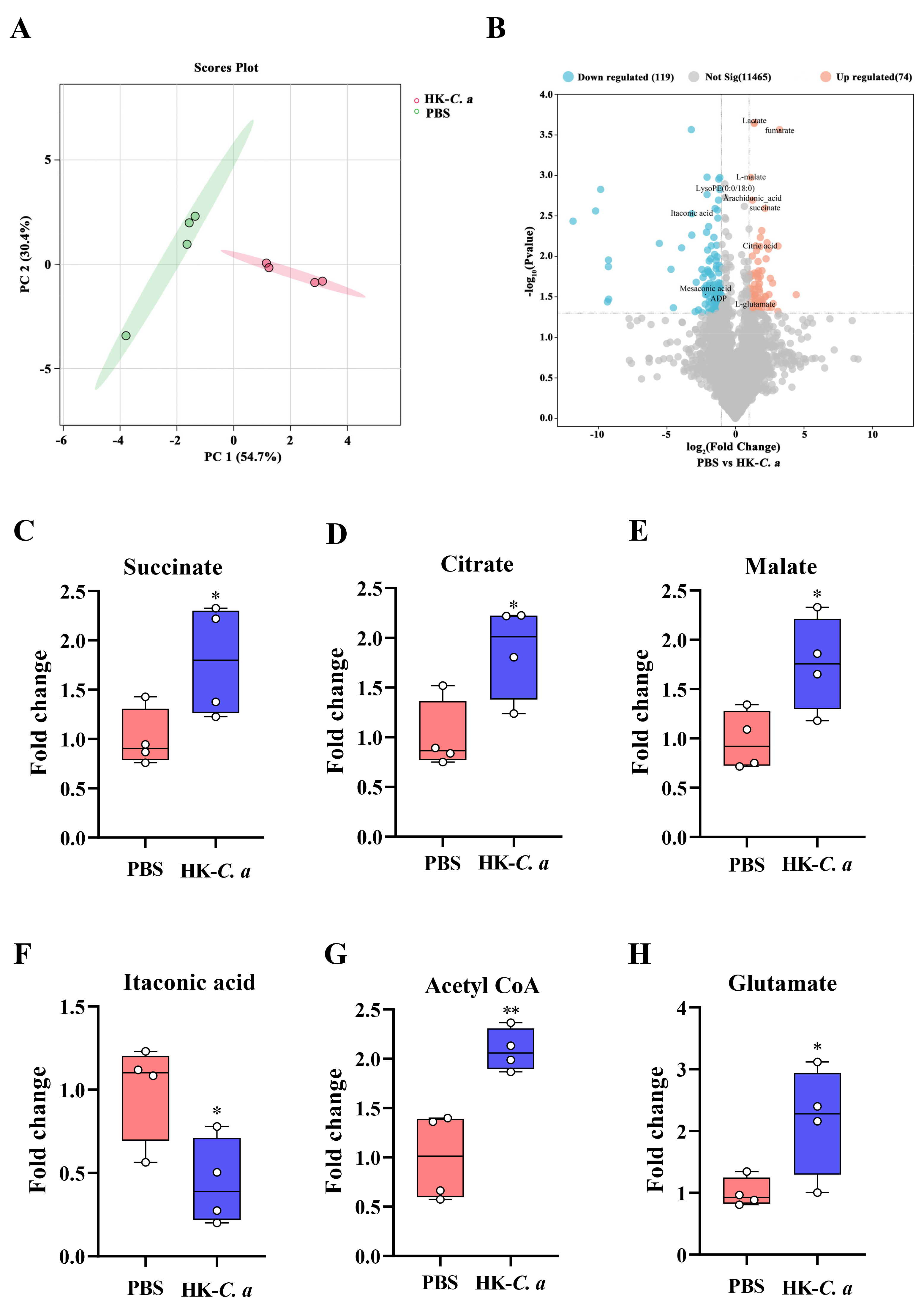

Supplement: S4 Fig — (A) Principal component analysis (PCA) score plot and (B) volcano plot of differential metabolites (p < 0.05) between PBS and HK-C. a-treated macrophages. (C-H) Box plot of the differential metabolites correlated with TCA cycle for PBS vs HK-C. a-treated macrophages. n = 4 biological replicates per group. *p < 0.05, **p < 0.01 indicate significant differences from each group. Student t test analysis of variance was performed and the data are presented as the means ± SEM. (TIF) [file ppat.1011918.s004.tif]

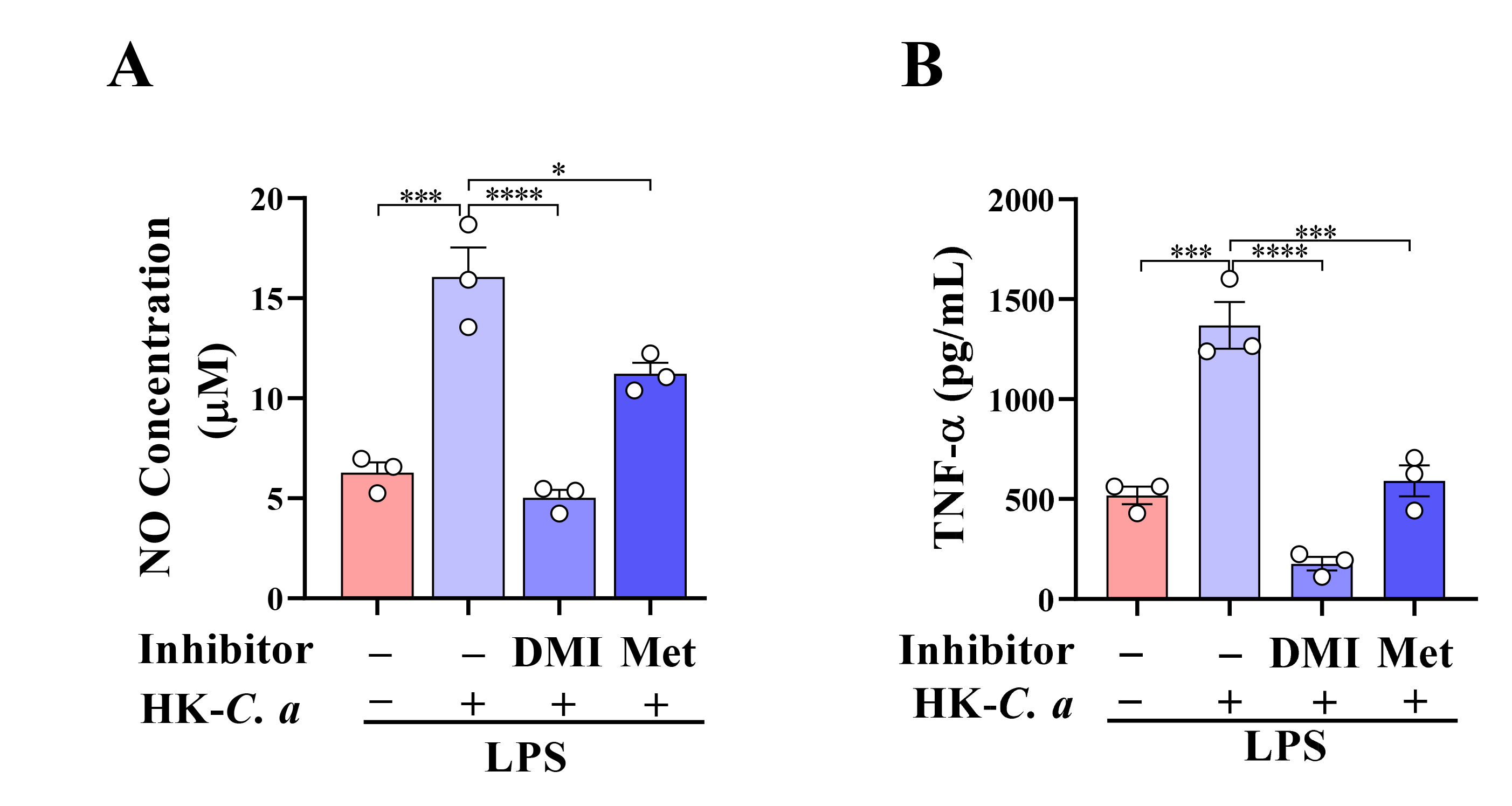

Supplement: S5 Fig — (A-B) Mouse peritoneal macrophages were trained with HK-C. a or left in culture medium for 24 h in the presence or absence of SDH inhibitor (DMI) or mTOR inhibitor (Met). After 5 days, cells were restimulated with LPS to determine NO (A) and TNF-α (B) production. The data are shown as means ± SEM, Data pooled from 3 independent experiments with 3 replicates per group. Significance was determined using a one-way ANOVA with a Dunnett’s multiple comparisons test; *p < 0.05, ***p < 0.001, ****p < 0.0001. (TIF) [file ppat.1011918.s005.tif]

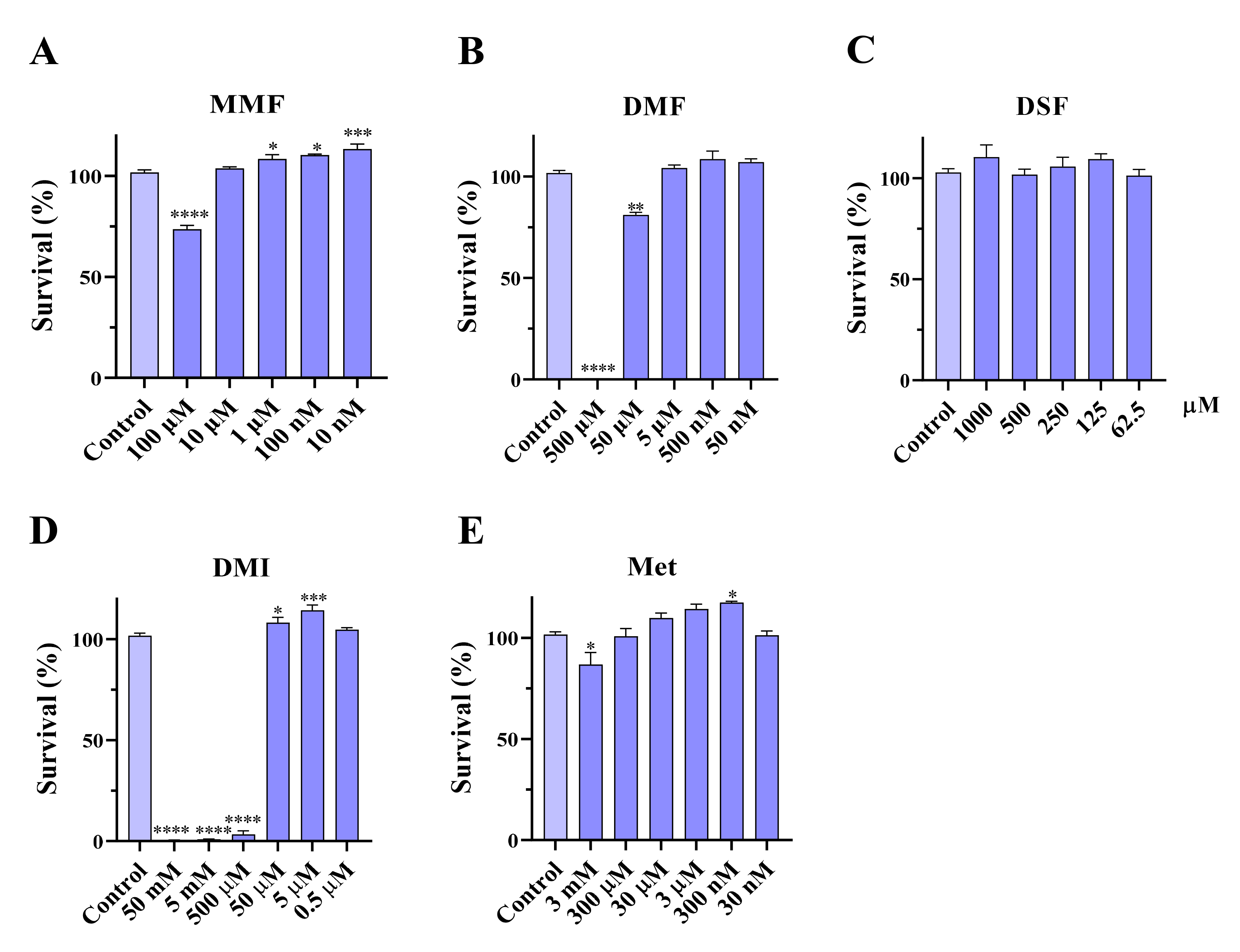

Supplement: S6 Fig — Peritoneal macrophages were treated with MMF (A), DMF (B), DSF (C), DMI (D), and Met (E) at different concentration conditions. Each data is presented as the means ± SEM. Significance was determined using a one-way ANOVA with a Dunnett’s multiple comparisons test; *p < 0.05, **p < 0.01, ***p < 0.001, ****p < 0.0001 vs Control. (TIF) [file ppat.1011918.s006.tif]

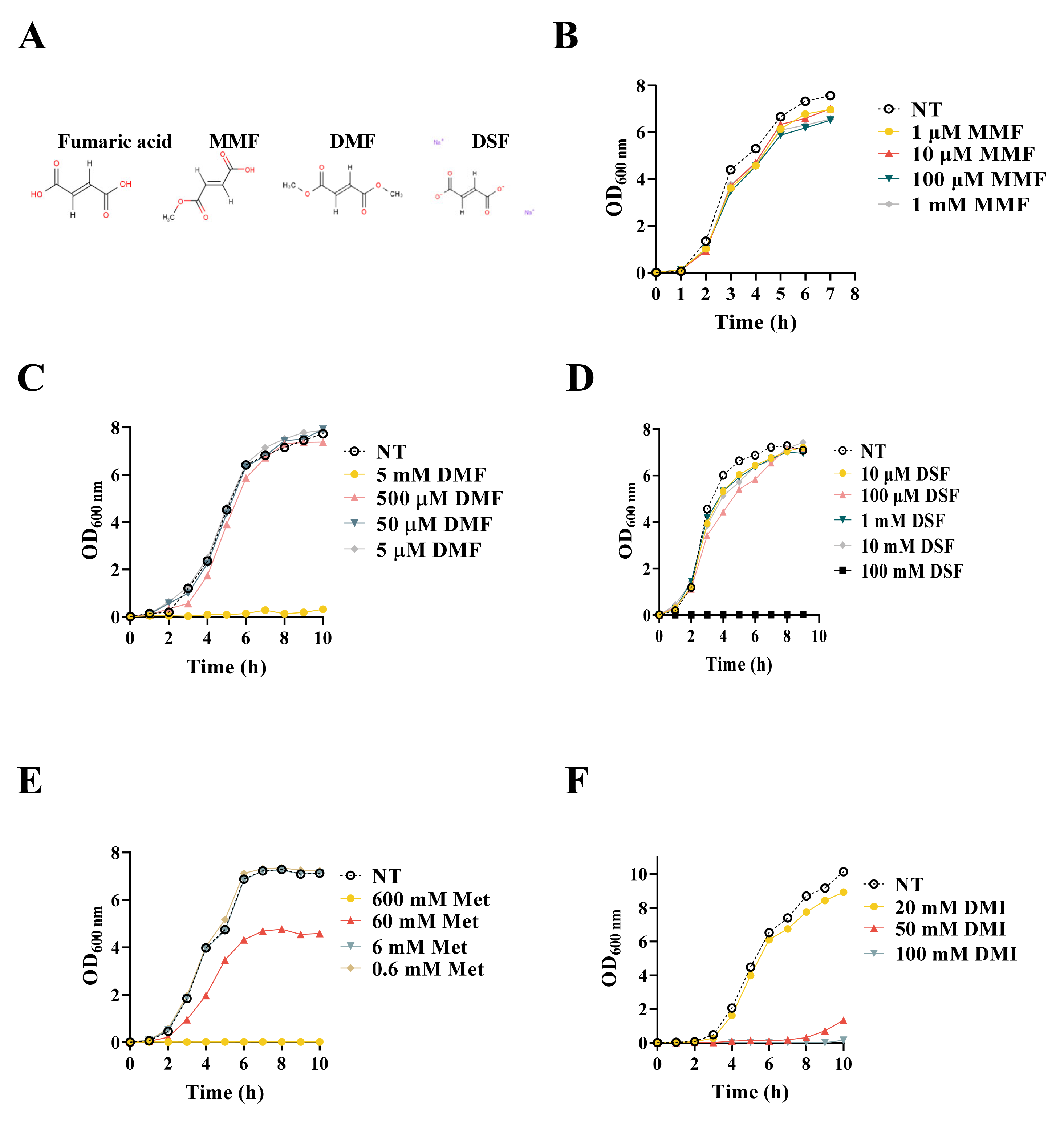

Supplement: S7 Fig — (A) Structural formula of fumaric acid and its derivatives. (B-F) The growth curves of S. aureus in exponential phase culture co-incubated with different concentrations of MMF (B), DMF (C), DSF (D), Met (E), and DMI (F). (TIF) [file ppat.1011918.s007.tif]

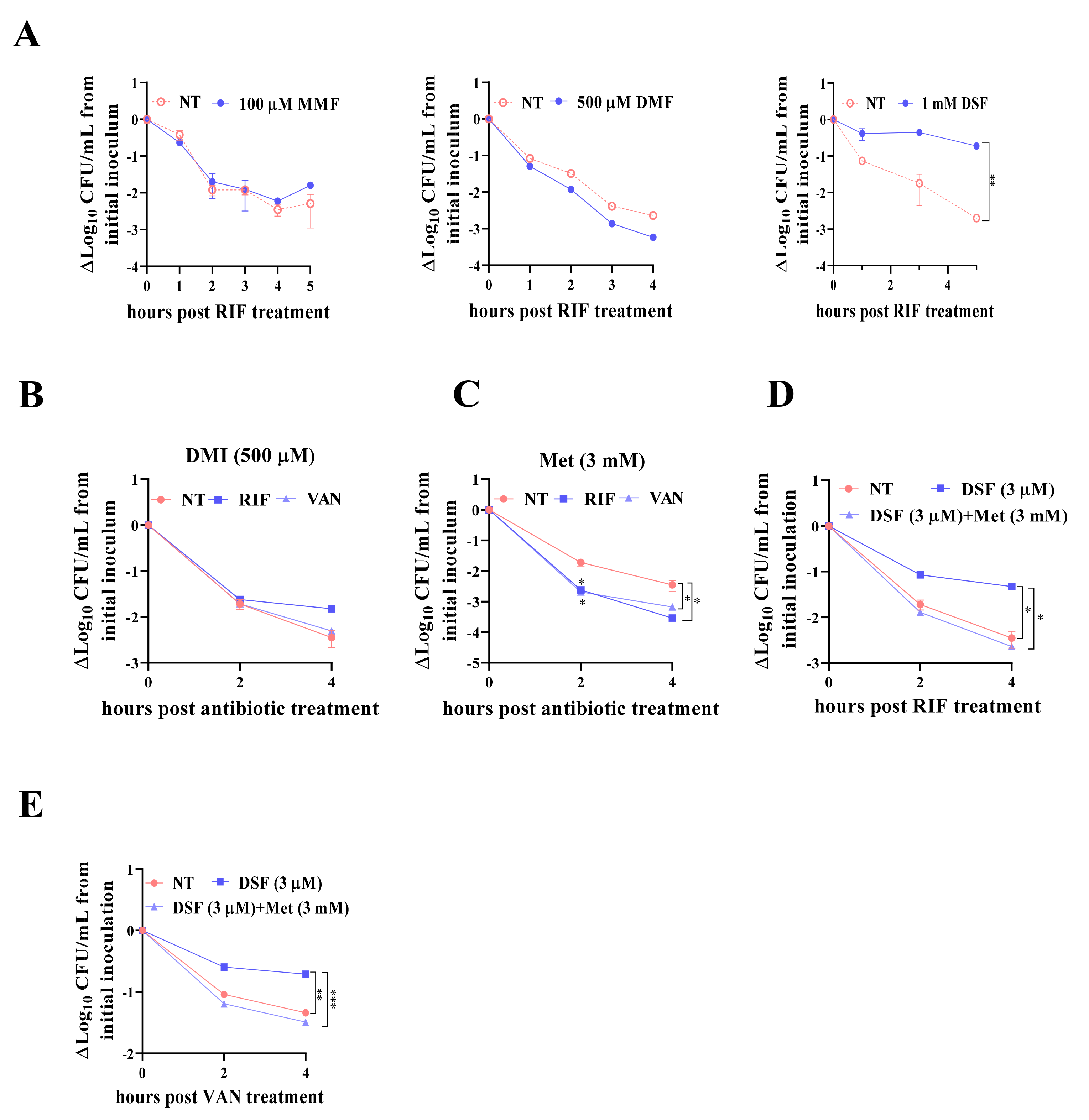

Supplement: S8 Fig — (A) Time-kill curves of S. aureus pre-incubated with MMF, DMF, and DSF followed by rifampicin treatment. (B-C) Fraction of surviving cells of S. aureus pre-treated with DMI (B) or Met (C) after secondary treatment with rifampicin or vancomycin for indicated times. (D-E) Fraction of surviving cells of S. aureus pre-treated with DSF and Met after secondary treatment with rifampicin (D) or vancomycin (E) for indicated times. Significance was determined using a two-way ANOVA with a Tukey multiple comparisons test; *p < 0.05, ***p < 0.001, ****p < 0.0001. (TIF) [file ppat.1011918.s008.tif]
