# Supplementary material for: Trained immunity in recurrent Staphylococcus aureus infection promotes bacterial persistence
Source: PLoS Pathog. 2024 Jan 19;20(1):e1011918. doi: 10.1371/journal.ppat.1011918 (PMC10798626; doi:10.1371/journal.ppat.1011918)
Supplement: S1 Table — (DOCX) [file ppat.1011918.s009.docx]

**S1 Table. Baseline characteristics of mastitis cows with *S. aureus* infections**

| **Covariate** | **Nontolerant strain (n = 61)** | **Tolerant strain**  **(n = 15)** | **P value** |
| --- | --- | --- | --- |
| Parity  (mean ± SEM) | 2.541 ± 0.16 | 3.133 ± 0.36 | 0.115 |
| Age (months,  mean ± SEM) | 55.31 ± 5.27 | 55.66 ± 2.87 | 0.956 |
| Past (90 d) infection | 27.86% | 80% | 0.0006 |
| Days in milk  (mean ± SEM) | 104.1 ± 15.17 | 111.9 ± 33.24 | 0.823 |
| Other diseases | 19.67% | 16.67% | 0.724 |

Abbreviations: SEM, standard error of mean. Other diseases include digestive disease, enteritis, displacement of the abomasum, retained fetal membranes, metritis etc. Data are analyzed by either one-way ANOVA followed by Bonferroni correction or Fisher’s exact test.
